# Multidisciplinary Clinical Approach to Cancer Patients with Immune-Related Adverse Events Induced by Checkpoint Inhibitors

**DOI:** 10.3390/cancers12113446

**Published:** 2020-11-19

**Authors:** Maria-Carlota Londoño, Maria Reig, on behalf of the RETOINMUNO Multidisciplinary Group

**Affiliations:** 1 Liver Unit, Hospital Clinic Barcelona, IDIBAPS, University of Barcelona, CIBERehd, 08036 Barcelona, Spain; 2 Liver Liver Cancer Group (BCLC), Liver Unit, Hospital Clínic Barcelona, IDIBAPS, University of Barcelona, CIBERehd, 08036 Barcelona, Spain

**Keywords:** immune checkpoint inhibitors, immune-oncology, immune-related adverse events, protocols, multidisciplinary approach

## Abstract

**Simple Summary:**

This manuscript revises the harmonised management of side effects related to the use of certain drugs to treat cancer called checkpoint inhibitors. These drugs activate immune responses against tumour cells and by doing so they may also induce immune responses against self-tissues leading to clinical manifestations that affect several organs and systems. The complexity of this scenario requires a multidisciplinary care, integrating several health professionals (specialized nurses, prescribers, and non-prescriber specialists) that consider all treatment options and develop an individual treatment plan for each patient aiming to guarantee the best patient care. The recommendations presented here are based on current guidelines for the management of organ-specific immune-related adverse events.

**Abstract:**

Immune-oncology is a major breakthrough in cancer treatment and has become the standard of care for a wide variety of solid organ malignancies. Unfortunately, manipulation of the immune system with checkpoint inhibitors may result in an immune-based attack of normal tissues which can lead to treatment discontinuation. These immune-related adverse events (irAEs) are diverse and affect several organs, constituting a new clinical challenge in the management of cancer patients. The complexity of this scenario requires a multidisciplinary approach that allows the early identification, diagnosis and treatment of specific irAE, ruling out other non-related adverse events. Hospital Clinic has a multidisciplinary team seeking to develop a coordinated strategy to facilitate the access of patients with suspected irAEs to specialised care resulting in harmonised management that guarantees the best patient care. The aim of the manuscript was to describe the current evidence on the management of irAEs reflecting a coordinated multidisciplinary approach to face this clinical challenge regardless of the immunotherapy indication.

## 1. Introduction

Cancer immunotherapy has become the standard of care for a wide variety of solid organ malignancies. It provides the unprecedented opportunity to treat, and in some cases, achieve long-term complete remissions of several previously untreatable cancers [1]. This occurs in the era of the “silver oncologic tsunami” and growing numbers of long-term cancer survivors with frequent comorbidities. Currently, the most commonly used approach is the administration of immune checkpoint inhibitors (ICPI). These drugs block proteins with critical function as negative regulators of T cell activation [2,3,4]. CTLA-4 suppresses the activation of T cells by out-competing with CD28 for the ligation with CD86 and CD80 of antigen presenting cells (APC). In addition, CTLA-4 expression in regulatory T cells (Tregs) can directly remove CD86 and CD80 from the surface of activated APC by trans-endocytosis [5]. Monoclonal antibodies against CTLA-4 (ipilimumab, tremelimumab) induce T cell activation and lead to a depletion of Tregs within the tumour. When PD-1 binds its receptor (PD-L1, expressed in tumour cells, epithelial cells, dendritic cells, macrophages and fibroblasts; or PD-L2, only expressed in APC), T cell activation is inhibited [6]. PD-1 (nivolumab, pembrolizumab, cemiplimab)/PDL-1 (atezolizumab, durvalumab, avelumab) axis blockade reverts these changes, and therefore, constitutes the pillar of current anti-cancer immunotherapy [7].

Different drugs are now approved for the treatment of multiple cancer types either in monotherapy or in combination with other agents as first-line treatment or when standard treatment has failed (Table S1). Nowadays, immunotherapy has demonstrated to achieve response in a subset of cancers, although it is still difficult to precisely determine which patients will benefit [8]. New strategies based on pharmacokinetic and pharmacodynamic monitoring, as a means of improving exposure and predicting individual response to ICPI, have not yet been fully evaluated to guide dose individualization [9].

Unfortunately, the manipulation of the immune system with these drugs can result in an immune-based attack on healthy tissues. Its consequences are diverse and can potentially affect every organ [7] constituting a new clinical challenge in the management of cancer patients. Anti-PD-1 and anti-CTLA-4 blockers present different mechanisms of action, acting on different sites and affecting distinct lymphocyte subtypes. Their concomitant use results in a higher incidence and broader spectrum of adverse events (AEs), also known as immune-related adverse events (irAEs). Some studies suggest that the development of irAE may be associated with better anti-tumour responses. However, this finding may differ between cancer types and specific drugs and there is still poor knowledge regarding that issue. The complexity of this scenario requires multidisciplinary care, which can be defined as an integrated team approach in which health professionals consider all treatment options and develop an individual treatment plan for each patient [10]. A multidisciplinary approach for cancer care has been recommended by cancer organizations, governments and societies since 1995 [11]. Traditionally, these groups are composed by expert clinicians from the different specialties involved in the care pathway of each tumour according to its primary location (medical and radiation oncologists, dermatologists, urologists, gynaecologists, haematologists, neurologists, hepatologists, pneumologists, gastroenterologists, endocrinologists, internists, rheumatologists, surgeons, radiologists, pathologists, intensive care specialists, specialised nurses, psychologists, etc.).

**Figure 1:**
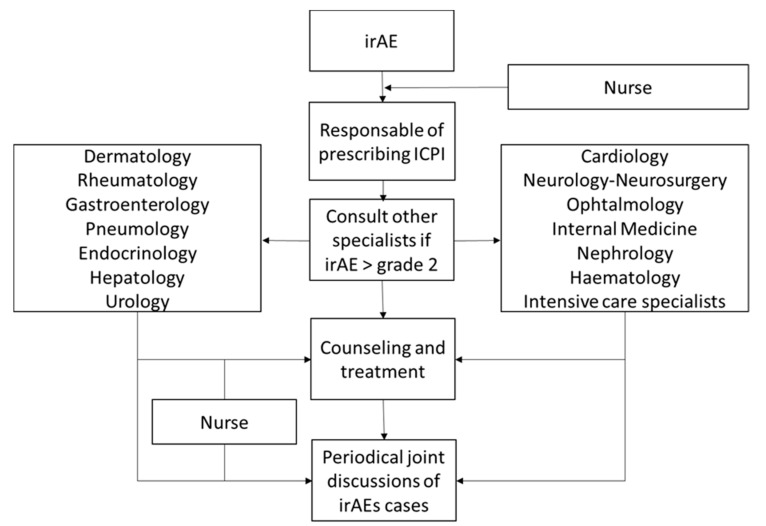
Workflow of the multidisciplinary approach to the management of immune-related adverse events (irAEs).

In 2018, our hospital created a multidisciplinary team seeking to develop a coordinated strategy to facilitate the access of patients with suspected irAEs to specialised care. As a result, harmonised protocols were designed to assure the best irAE management and provide state of the art medical care for both conventional practice and research (Figure 1). Here, we summarise the current evidence and propose a coordinated strategy to face the current clinical challenge posed by cancer immunotherapy-irAEs.

## 2. Coordinated Management of irAEs

### 2.1. Skin

Dermatological toxicity is considered the most frequent ICPI-induced AE, which is usually the first to appear (even within the first weeks of treatment) and is rarely severe. As a result, these AEs usually do not lead to ICPI treatment modification [12,13]. However, dermatological complications can develop at any time during treatment, even after discontinuation. On rare occasions, serious immune-mediated dermatosis may appear—the so called severe dermatological adverse event (SDAE) [14,15]. Therefore, early dermatological evaluation is crucial to ensure accurate recognition and management in early stages of the toxicity. That is the case of potential life-threating syndromes such as toxic epidermolytic necrolysis (TEN), the Stevens–Johnsons syndrome (SJS), drug rash with eosinophilia and systemic symptoms (DRESS) or acute generalised exanthematous pustulosis (AGEP). The most important criteria to suspect a potential severe dermatological AE are summarised in Figure S1.

Approximately 1 out of 3 patients treated with ICPI develop a dermatological irAE; 45% with anti-CTLA4, 35% with anti-PD1 and rising two-fold when used in combination (anti-CTLA4 + anti-PD1). Less than 5–8% of patients develop grade 3 to 4 dermatological toxicity (around 2–3% with monotherapy and 5% with combined therapy).

**Figure 2:**
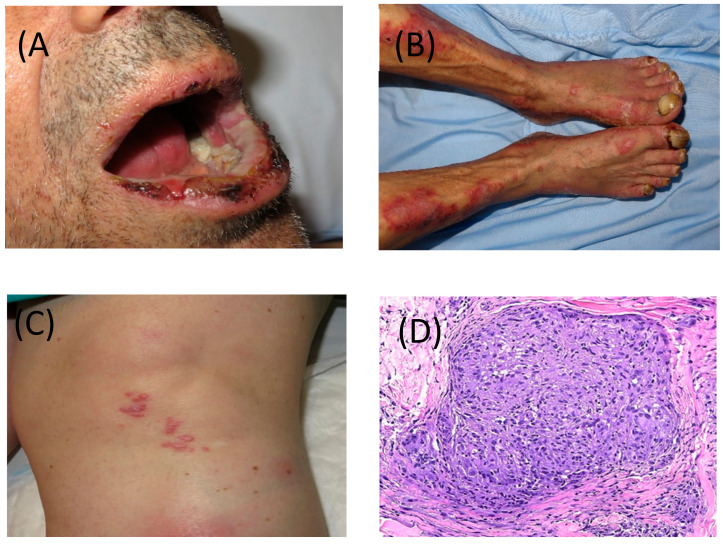
(**A**) Severe and erosive lichen planus-like lesions with involvement of skin and nails and (**B**) of mucosa, after 3 cycles of nivolumab. (**C**) Cutaneous involvement of sarcoid-like granulomatous lesions developed after cessation of pembrolizumab. (**D**) Granulomatous lesion in skin biopsy, magnified 40 times.

Skin rash is the most frequent irAE (15% in anti-PD1, 25% in anti-CTLA4 and 40% in combination); erythematous unspecific self-limited exanthema can appear very early, but the most specific irAE consists of a lichenoid exanthema. It can present the same features as an idiopathic lichen planus rash (Figure 2A,B) on the skin and/or mucosae or may show more diffuse involvement such as that of drug-induced lichenoid. Vitiligo-like depigmentation, which presents with hypo-pigmented or white macules usually on the trunk and sun-exposed areas, can be observed especially in melanoma patients treated with ICPI (less than 10%). This side effect has been most strongly associated with better response to therapy and improved outcomes in melanoma patients; however, prospective, long-term follow-up studies are necessary to confirm this association [16,17]. Vitiligo-like macules do not need specific treatment. Pruritus was initially reported in about 25–35% of cases, although it is probably less frequent, at least at a high enough grade to require specific management which is estimated to occur in about 15% of cases. However, up to 2% of cases can reach grade 3–4 pruritus in which the patient cannot tolerate ICPI therapy.

Several types of dermatosis can be induced or exacerbated during or after ICPI therapy. Most involve immune-mediated disorders probably in predisposed individuals. Some of these diseases include psoriasis, autoimmune blistering diseases and connective tissue disorders, Grover’s disease, alopecia areata, xerosis, urticaria, among others. In view of this wide differential diagnosis, dermatological consultation is strongly recommended. Granulomatous disorders can also be early detected on the skin, and the most frequent organ involved is the lung and hiliar lymph nodes. Sarcoid-like granulomas can pose a diagnostic challenge to be differentiated from metastatic disease (Figure 2C,D).

Management of these skin diseases depends on the definite dermatological diagnosis, but the treatment of most dermatological irAEs is usually based on symptomatic oral therapy for pruritus with antihistamine drugs, and topical and/or oral corticosteroids, not requiring ICPI modifications. Treatment recommendations are based on the Common Terminology Criteria for Adverse Events (CTCAE) classification, generally based on the extension of the rash on the body surface as well as patient tolerability. Table 1 shows the protocol for skin rash [18].

Other immune-modulator drugs, such as azathioprine, cyclosporine or mofetil mychophenolate, may be of benefit in cases presenting irAEs, and individual management must be discussed. In the case of the development of SDAE, early vital and general support by a referral centre is essential, and the ICPI usually has to be definitely discontinued.

### 2.2. Gastrointestinal Tract

Among all the irAEs associated with ICPI, gastrointestinal (GI) toxicity is one of the most frequent and severe, being diarrhoea and colitis the most prevalent [19]. Colitis must be suspected when diarrhoea is accompanied by abdominal pain and/or rectal bleeding. More severe cases may resemble inflammatory bowel disease (IBD). Enteritis, esophagitis, necrotizing gastritis and pancreatitis are rare but have also been reported. Microscopic colitis has also been described, being histological diagnosis mandatory for the establishment of diagnosis.

The incidence of diarrhoea and colitis related to CTLA-4 inhibitors is higher at 35% and 10%, respectively and more severe compared to that induced by PD-1 blockade with 20% and 1.6%, respectively [20,21]. The frequency and severity of GI toxicity is dose-dependent and increases when the two drugs are given in combination. The median time to symptom onset is 8 weeks for ipilimumab compared to 3–6 months for nivolumab [22]. The severity of GI toxicity is established in four categories according to the CTCAE (Common Terminology Criteria for Adverse Events) classification. This scale considers symptoms including diarrhoea, abdominal pain, blood or mucus in the stools, incontinence and fever. In the most serious cases life-threatening conditions such as haemorrhage, perforation and toxic megacolon may appear. Colonic perforation has been described in up to 6% of patients treated with ipilimumab.

The diagnostic algorithm depends on the severity of the symptoms. In all cases, the initial workup should include a complete blood count, serum electrolyte profile, C reactive protein (CRP), renal and thyroid function, and stool cultures to rule out infections (bacteria and *Clostridium difficile*). In moderate to severe cases, an abdominal CT and colonoscopy are recommended. Two distinct patterns of clinical and radiographic manifestations of ICPI-related colitis have been reported. The most common is a diffuse unspecific colitis pattern, characterised by mild diffuse bowel wall thickening or colonic distension with prominent pericolonic vessel engorgement. Another less common pattern is segmental colitis associated with sigmoid diverticula. In these cases, both colonic wall thickening and perienteric inflammation are more evident, resembling an infectious diverticulitis, but patients usually have a paucity of systemic symptoms [23,24]. Full colonoscopy with biopsies is advisable since sigmoidoscopy may underestimate the extent and severity of the lesions (up to 8% of patients may have lesions limited to the ascending colon). Around 75% of patients present extensive colitis with a continuous pattern of inflammation in 45–79% [25]. Endoscopic findings may resemble infectious conditions and IBD (erythema, loss of vascular pattern, friability, ulcers and mucosal bleeding). Severe endoscopic lesions and pancolitis correlate with the need for rescue therapy with infliximab [26,27]. Biopsies are mandatory to establish an adequate diagnosis (rule out the presence of cytomegalovirus). In cases of normal appearance of the mucosa, biopsies should also be obtained in order to rule out microscopic colitis.

Mild cases of diarrhoea can be managed with symptomatic treatment including a low-fibre diet or anti-diarrhoeal drugs such as loperamide and oral fluids. Immunotherapy can be continued in mild cases. In moderate or mild cases not responding to symptomatic treatment, oral corticosteroids (1 mg/kg) must be started and tapered within 8–12 weeks. All severe cases must receive intravenous corticosteroids (1–2 mg/kg) together with supportive measures. In moderate and severe cases, immunotherapy must be discontinued until the condition has resolved. Although the response rates to corticosteroids are high (80%), a non-negligible proportion of patients (20%) present steroid refractoriness. Early identification and adequate treatment of this situation (persistent elevated CRP and/or blood in the stools after 3–5 days of treatment) is crucial to reduce morbidity and mortality. Patients not responding to intravenous corticosteroids within 3–5 days should be treated with infliximab (5 mg/kg) [28,29,30]. A single dose is often enough for improving symptoms, although a second or even third dose may be needed 2 and 6 weeks later, respectively. Vedolizumab, a monoclonal antibody blocking α4β7 integrin, has recently shown to be effective in a small series of patients with steroid-dependent, steroid-refractory and infliximab refractory immune-mediated colitis [31,32]. In extremely severe cases, patients may require emergency subtotal colectomy (megacolon, colonic perforation) (Table 1).

### 2.3. Lung

Immune-mediated pulmonary toxicity, known as pneumonitis and defined as local or diffuse inflammation of the lung parenchyma secondary to treatment [33], is a rare adverse effect (2.7%) and may be severe in some cases (0.8%) [34]. Pneumonitis (of any degree or severity) is more prevalent in patients with non-small cell lung carcinoma as compared to other tumours such as melanoma (4.1% vs. 1.6% and 1.8% vs. 0.2%, respectively) [35]. Similar to other irAEs, pneumonitis is more frequent and severe in patients receiving combined treatments (6.6% vs. 1.6% in patients treated with only one ICPI) [35]. In addition to the typical pulmonary involvement due to pneumonitis, other immune-mediated pulmonary toxicities such as tracheobronchial involvement, sarcoid reactions and pleural effusion may be presented, albeit less frequently. The clinical manifestations reported in the different studies consist of non-specific respiratory symptoms such as dyspnoea (53%) and cough (35%), although they can also manifest with fever (12%) and chest pain (7%). AEs may be asymptomatic in 33% of patients [12]. Pneumonitis is classified according to the adaptation of the CTCAE clinical criteria [12]: grade (G)1 (asymptomatic, only associated radiological changes), G2: (mild or moderate symptoms), G3 (severe symptoms, need for oxygen therapy) and G4 (respiratory failure, need for intubation or tracheostomy). The clinical guidelines of the European Oncology Society (ESMO) [36] recommend the following complementary tests for suspected immune-mediated pneumonitis: (1) Conventional chest X-ray, as the initial diagnostic test on suspicion of any degree of pneumonitis; (2) thoracic high-resolution computed tomography, indicated in pneumonitis ≥ G2; (3) bronchoscopy and bronchoalveolar lavage with microbiological and cytological study in ≥ G2 pneumonitis in order to rule out other infectious or concomitant processes of pharmacological toxicity. In G1 pneumonitis, symptomatic treatment and delay of ICPI treatment and revaluation according to evolution is recommended. In G2 pneumonitis, it is advisable to suspend ICPI treatment and initiate oral or intravenous corticosteroid treatment at a dose of 1 mg/kg per day. In G ≥3 pneumonitis, immunotherapy treatment should be permanently discontinued and intravenous corticosteroids should be initiated at a dose of 2–4 mg/kg/day (Table 1).

### 2.4. Rheumatic and Systemic Autoimmune Syndromes

There is an increase of the number of reports of rheumatic and systemic autoimmune syndromes induced by ICPI, due to increased usage of ICPI as well as better recognition of these new associations [37]. Overall, according to some recent reports, these syndromes occur in around 5–10% of patients receiving ICPI [38,39,40,41,42,43], although randomised clinical trials may sometimes underreport and/or misclassify these syndromes as musculoskeletal disorders. These irEAs can be grouped into four different categories: (1) inflammatory arthritis, (2) non-inflammatory arthralgias, (3) polymyalgia rheumatic-like (PMR) syndromes and (4) a miscellaneous group of systemic or localised disorders including vasculitis, sicca symptoms and scleroderma- and sarcoid-like reactions.

The time to onset of rheumatic syndromes induced by ICPI is variable. In general, inflammatory arthritis usually appears around 8 weeks after initiation of therapy, but some patients may develop arthritis even several months after treatment withdrawal [37,39,43,44].

**Figure 3:**
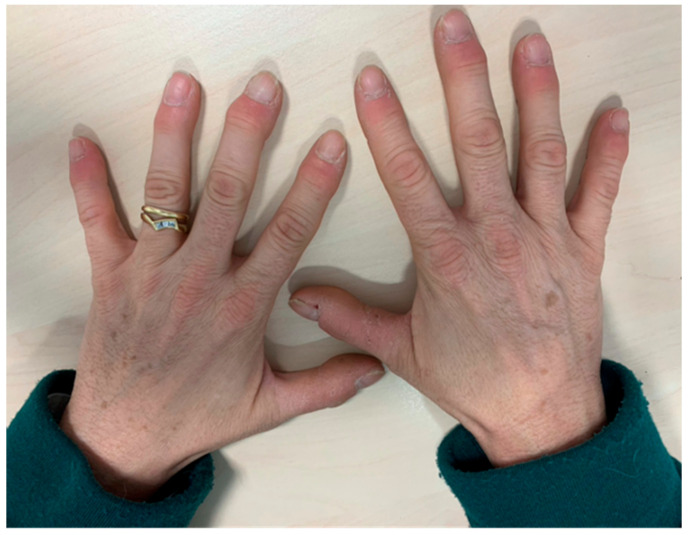
Symmetric distal swelling of interphalangeal joints with painful erythema in periarticular structures.

The symptoms of patients who develop psoriatic arthritis-like syndromes are heterogeneous, including nail involvement, distal interphalangeal arthritis, enthesitis and less frequently, axial inflammatory pain (Figure 3). Patients with PMR due to ICPI may present with a mild increase of acute phase reactants, and response to corticosteroids might be unsatisfactory compared to the classic syndrome [37].

Although much more uncommon than articular manifestations, case series of sicca syndrome and sarcoid-like reactions have been reported. Moreover, cases of well-defined systemic vasculitis, such as giant cell arteritis or antineutrophil cytoplasmic antibody (ANCA)-associated vasculitis, and sporadic cases of systemic erythematous lupus, anti-phospholipid syndrome and scleroderma reactions have also been identified during ICPI treatment [37,45].

Since immune phenomena are predominantly T-cell mediated, autoantibodies are not usually seen in patients with rheumatic syndromes related to ICPI. However, it is important to bear in mind that some autoantibodies (anti-nuclear antibodies, anti-neutrophil cytoplasmic antibodies, rheumatoid factor or antiphospholipid antibodies) are not uncommon at low titres in the elderly population with active neoplasms [37,39].

Given the increase in the use of ICPI for different solid and haematological neoplasms, it is not uncommon for some patients with underlying rheumatic diseases to receive ICPI. Clinical flares are not infrequent in these patients, but the use of ICPI is not contraindicated. An interesting fact is that cancer patients presenting rheumatic manifestations or other irAEs are likely to respond to ICPI treatment (85%) and may have a better oncologic prognosis than those patients without irAEs [46,47].

The treatment of these rheumatic syndromes must be based on shared decision making between the rheumatologist or another specialist in autoimmune diseases and the referring specialist prescribing ICPI treatment. Recently, the American Society of Clinical Oncology (ASCO) proposed a guideline for the diagnosis, classification and treatment of irAEs of ICPI including rheumatic syndromes [40]. Low to moderate doses (0.5 mg/kg/day) of oral corticosteroids are the standard treatment for inflammatory rheumatic side effects related to ICPI.

Non-steroidal anti-inflammatory drugs (NSAIDs), analgesics and/or physiotherapy are recommended for non-inflammatory conditions. There is usually no need to stop or even modify ICPI treatment. In some cases, with severe polyarthritis, intravenous methylprednisolone pulses may be needed for a short period of time (125 mg/day). In a small proportion of patients with persistent synovitis, steroid-sparing treatments are recommended to minimise side effects. The therapy of choice in these cases is disease modifying anti-rheumatic drugs (DMARDs) used in inflammatory arthropathies usually involving oral or subcutaneous methotrexate at standard doses (10–20 mg per week). In some severe cases, discontinuation of ICPI therapy is necessary. In addition, there are some anecdotal reports of biologic DMARD use in patients with ICPI-induced inflammatory arthropathies, mainly anti-tumour necrosis factor (TNF) therapies. Systemic vasculitis (giant cell arteritis or ANCA-associated vasculitis) should be treated as classic entities (corticosteroids ± immunosuppressant drugs), and ICPI must usually be stopped. Management of sicca syndrome and sarcoid-like reactions is conservative with symptomatic treatment, and ICPI can usually be continued.

### 2.5. Endocrine

Endocrine toxicity is one of the most frequent toxicities associated with both anti-CTLA-4 and anti-PD1/anti-PDL-1. Although the different types of ICPI have been associated with both thyroid disorders and hypophysitis, the first is generally correlated with anti-PD1/anti-PDL-1 and the second to anti-CTLA-4. The onset is around 7–8 weeks, although it can appear after the first dose and years after ICPI discontinuation. Combined therapy with anti-PD1/anti-PDL-1 and anti-CTLA-4 is associated with a higher incidence of endocrine toxicity, early onset (during the first month) and polyglandular and severe presentations (G3-G4) [48].

Little evidence is available on susceptibility markers for endocrine irAEs associated with ICPI. Therefore, the best approach is: (1) to closely monitor ICPI-treated patients for clinical and analytical signs of endocrine dysfunction before and during ICPI treatment (Figure S2), and (2) to treat irEAs early [49]. Notably, the use of corticosteroids at high doses does not modify the grade of glandular involvement, the recovery of which depends on the type of affected gland, and no prognostic markers have been identified yet. Moreover, endocrine toxicity seems to be correlated with the efficacy of ICPI and a higher survival rate, but the evidence on this issue is still limited [50,51].

Thyroid disorders are the most frequent endocrine irAEs, especially in association with anti-PD-1/anti-PDL-1 (4–40%) [52,53]. In general, the clinical presentation consists of silent thyroiditis diagnosed in blood tests with elevated free-T4 and suppressed thyroid stimulating hormone (TSH). Thyroiditis may progress to transient thyrotoxicosis, euthyroidism or hypothyroidism (up to 30%). Sporadic cases of Graves’ disease and thyroid ophthalmopathy have been described in association with anti-CTLA-4 treatment. Presentation is usually G1–G2 and thyroid storm or myxoedema are extremely rare. Previous thyroid disease or an endocrinological thyroid-related irAE are not contraindications for ICPIs. In thyrotoxicosis or severe hypothyroidism, ICPI should be withheld. Aetiological diagnosis may require anti-antiperoxidase (thyroid peroxidase (TPO)) antibodies for hypothyroidism and anti-TSH receptor antibodies, thyroid scintigraphy and/or doppler ultrasound in hyperthyroidism. When the patient presents with TSH >10 mIU/L or between 5–10 mIU/L with positive anti-TPO or clinical symptoms of hypothyroidism, replacement treatment is required (starting with 0.8 mcg/kg/d, 25–50 mcg/d in older people or those with comorbidities, mainly cardiac, and scaling up to 1.6 mcg/kg/d approximately). In asymptomatic thyrotoxicosis, silent monitoring can be proposed; in symptomatic thyrotoxicosis beta-blockers are recommended and in Graves’ disease, anti-thyroid drugs should be associated. Corticosteroid therapy should only be considered in clinically severe cases of thyrotoxicosis. Recovery of thyroid function is potential but unpredictable (up to 75% in hyperthyroidism and up to 15% in hypothyroidism) [48,49].

Hypophysitis is more frequent in patients receiving anti-CTLA-4 (up to 0–17%), especially in males around 60 years-old and 2–3 months after beginning ICPI [52,53]. The clinical presentation may be related to an increase in gland volume and compression symptoms (headache and/or visual disturbances) or may be related to hormonal dysfunction (asthenia, decreased libido and amenorrhoea). The gonadal and thyroid axes are almost always affected (85–100%) but with a high probability of recovery. On the contrary, the corticotropic axis is less affected (50–73%), but the probability of recovery is very low (0–15%). Diabetes insipidus is rare. When hypophysitis is suspected, blood electrolytes and hormone tests are recommended before treatment (Figure S3). Pituitary magnetic resonance imaging (MRI) should also be performed to rule out pituitary metastasis or haemorrhages, taking into account that a normal image does not rule out the diagnosis of hypophysitis [49]. On the other hand, anti-PD-1/anti-PDL-1 drugs have been associated with an isolated reduction in adrenocorticotropic hormone (ACTH) levels, which appears around the 7th month of treatment with high variability. Symptoms of hypocortisolism are more frequent with anti-PD-1/anti-PDL-1 than with anti-CTLA-4: headache is rare, and hyponatremia is more prevalent. MRI is generally normal [54]. The management of hypophysitis is summarised in Figure S3 [49].

Other less frequent endocrine irAEs (insulitis, pancreatitis, and adrenalitis) are described in Text S1.

### 2.6. Liver

Hepatotoxicity due to the use of ICPI has been described in 3 to 9% of patients treated with anti-CTLA4, being of 1 to 4% in patients receiving anti-PD1, and 18% in those treated with a combination of both drugs [55,56]. Severe hepatitis (grade 3 or 4) occurs in approximately 3.5% of patients [24]. Liver toxicity generally manifests within the first 2 months of therapy, but it can also appear up to 6 months after starting ICPI treatment [57]. Most patients are asymptomatic, being diagnosed within the context of liver test abnormalities. However, it is important to bear in mind that immunotherapy is the cause of the increase in liver enzymes in only 16.7% of patients undergoing ICPI treatment [58].

During evaluation of patients with increased liver enzymes during ICPI treatments, it is important: (1) to determine the severity of liver dysfunction using the CTCAE scale in addition to parameters of liver synthesis (international normalised ratio (INR) and albumin), and (2) to rule out other causes of increased liver enzymes, including alcohol consumption, concomitant medications (drug-induced liver damage), use of herbal products or over-the-counter medications, viral hepatitis (A, B, C, E), idiopathic autoimmune hepatitis, hepatic or portal vein thrombosis, non-alcoholic fatty liver disease or progression of cancer [40,59]. A liver biopsy should be considered in patients with grade 3 or 4 hepatitis to evaluate the degree of liver inflammation, and to rule out other causes of liver disease [57]. However, the performance of liver biopsy should not delay treatment in patients with liver dysfunction (bilirubin >2.5 mg/dL and INR >1.5).

Less frequently, the bile duct can be the target of the ICPI-induced liver damage. The clinical manifestations are similar to those of patients with obstructive cholangitis (fever, abdominal pain and jaundice). Liver tests are characterised by an increase in alkaline phosphatase and γ-glutamyl transferase. MRI cholangiography shows bile duct dilation and hypertrophy [60].

Treatment of liver toxicity is based on the severity (Table 1). In patients with grades 1 and 2 hepatitis, monitoring is essential and withholding ICPI therapy is necessary in those with grade 2 hepatitis. In patients with more severe liver involvement (grades 3 or 4), ICPI treatment discontinuation is mandatory, and corticosteroid therapy (0.5–1 mg/kg/d) should be administered to patients with grade 4 liver toxicity, and those with grade 3 and signs of liver dysfunction, severe inflammation in the liver biopsy or worsening of liver tests [24,59,61,62].

### 2.7. Nervous System

Neurologic AEs in patients treated with IPCI are uncommon but are becoming increasingly recognised. They are usually mild (grade 1–2), manifesting with non-specific and transient symptoms (headache, dizziness, paresthesia), but in some cases neurologic complications may be severe (grade 3–4) and the spectrum of symptoms highly heterogeneous (aseptic meningitis, encephalitis, posterior reversible encephalopathy syndrome, cerebellitis, myelitis, mononeuritis, polyneuropathy and myasthenia). Overall, the reported frequency is 4% for anti-CTLA4, 6% for anti-PD1 and 12% when the two treatments are combined. However, the frequency of moderate-severe events is less than 1% for all types of ICPI [63,64]. Neurologic irAEs usually appear during the first 4 months, with a median of 6 weeks after treatment initiation, but may develop later. The clinical course may evolve rapidly and lead to severe disability or death. Early identification is crucial since the symptoms may be reversible with adequate diagnosis and treatment [65]. Myasthenia and encephalitis are associated with anti-PD1 whereas other neurologic AEs such as meningitis or Guillain–Barré-like syndrome are associated with anti-CTLA-4. Myasthenia has an early onset, and concurrent myocarditis and myositis are frequent, with a high fatality [66]. In patients with cancer the use of ICPI can potentiate paraneoplastic mechanisms (an immune response that reacts with common antigens expressed by tumoural and neural cells). Recognition of classical paraneoplastic neurological syndromes (limbic encephalitis, encephalomyelitis, cerebellar degeneration, sensory neuronopathy, enteric neuropathy or Lambert–Eaton myasthenic syndrome) or the detection of onconeural or neuronal-surface antibodies may have implications in patient treatment and outcomes [67]. Treatment with anti-PD1 or anti-PD-L1 has been shown to worsen or trigger paraneoplastic syndromes associated with a very severe form of encephalitis [68].

After ruling out cancer progression and infectious or metabolic causes of symptoms in patients with suspected neurologic irAE, the consultant neurologist can help to characterise the neurologic involvement and guide the additional work-up and management. The diagnostic algorithm depends on the profile of symptoms and may include brain and spine MRI (to differentiate from stroke, leptomeningeal spread or metastatic disease), analysis of cerebrospinal fluid (typically shows elevated protein levels with lymphocytic pleocytosis), electroencephalogram, electromyogram and antibody studies. In severe cases, ICPI should be discontinued [69]. Treatment strategies are shown in Table 1, and in most cases include corticosteroids, intravenous immunoglobulins or plasma exchange, and cyclophosphamide or rituximab (in refractory cases or patients with proven paraneoplastic disease). Infliximab, tocilizumab, mycophenolate, cyclosporine and natalizumab have been used in anecdotal reports.

### 2.8. Eye

The eye is an immune-privileged site and it is known that excessive immune response is suppressed by local and systemic mechanisms [70]. The ocular side effects related to ICPI are generally immune-related. Their incidence is approximately 1% [71,72,73] and they usually appear between 6 to 12 weeks after starting ICPI therapy [72]. Ocular side effects have most frequently been reported with anti-CTLA4, and comparatively more frequent with anti-PD1 than with anti-PDL-1 [71]. The association of multiple ICPI increases the risk of adverse ocular effects [71,74,75]. They may affect different parts of the visual system (orbit and ocular adnexa, ocular surface, retina, uveal tract, extraocular muscles and optic nerve) and are usually associated with other systemic adverse effects [71,72]. Prompt detection is mandatory since this can prevent structural damage with irreversible vision loss.

Ophthalmic side effects most frequently present as dry eye (1–24%) [71,76,77] and uveitis (1%) [71]. Dry eye can be managed with artificial tears or topical cyclosporine in severe cases [73,74]. Uveitis usually affects the anterior segment (anterior uveitis), but it can also affect the posterior segment (posterior uveitis), mimicking autoimmune uveitis (including Birdshot disease, Voght–Koyanagi–Harada syndrome) or severe retinal vasculitis [73,78,79,80]. In most cases, posterior uveitis needs systemic or intravitreal corticosteroids, intravenous immunoglobulin (IVIG) and more potent immunosuppression is needed in severe cases. The side effect most frequently observed involving the orbit and adnexa is myasthenia gravis (MG), which can produce diplopia and/or ptosis and can be associated with systemic symptoms such as respiratory distress. This complication can be effectively managed with systemic corticosteroids and in some cases, plasma exchange or IVIG is needed [81,82].

Ocular side effects rarely oblige ICPI treatment discontinuation or delay, although this may need to be considered in cases with severe visual loss or the risk of loss. However, ocular side effects are often associated with systemic side effects that can dictate the course of the treatment (Table S2).

### 2.9. Heart

Cardiotoxic effects of ICPI are infrequent but may be potentially serious. According to recent registers and meta-analyses, the incidence is 1–2% [83]. The onset is usually early after exposure to ICPI, with a median of 65 days (equivalent to 3 cycles), although it may appear after the first dose [84]. The manifestations are varied [85] and include: myocarditis, conduction abnormalities, coronary disease, non-inflammatory left ventricular dysfunction or pericarditis with or without pericardial effusion. Myocarditis is usually of autoimmune type mediated by T lymphocytes and is characterised by elevated troponin levels and systolic ventricular dysfunction in up to half of the cases [86]. Elevation of troponin values and the appearance of symptoms are fundamental to decide whether immunotherapy should be temporarily or permanently suspended and immunosuppressive treatment initiated. Arrhythmic disorders caused by diffuse or direct myocardial inflammation of the His-Purkinje system include first, second or third degree atrioventricular blocks and supraventricular or ventricular arrhythmias. Coronary disease can be manifested by the rupture of atherosclerotic plaque or due to coronary vasculitis or vasospasm. Systolic ventricular dysfunction can be presented in the form of dilated cardiomyopathy or Tako–Tsubo syndrome and occurs with elevation of natriuretic peptides as well as contractility abnormalities in cardiac imaging tests. In case of cardiac tamponade, urgent pericardiocentesis is needed. The global management of immune-mediated cardiotoxicity should be assessed jointly among the specialists involved and includes: (1) consider interrupting therapy (usually transiently), (2) initiating cardiological therapies (drugs, cardioversion, pacemaker implantation, pericardiocentesis, ventricular assist devices) and (3) initiation of immunosuppression (methylprednisolone as first option). Table S3 shows the main cardiac irAEs and recommendations.

### 2.10. Muscle

Previous data suggest that PD1/PDL1 inhibitor-associated myopathy may present with a wide range of clinical manifestations and degrees of severity including MG [81,87,88], necrotising myopathy [89,90,91] or even lethal cardiomyopathy [92,93,94,95]. The identification of new neuromuscular symptoms in patients receiving anti-PD1 antibodies may be a challenge, as these patients may be at greater risk of developing muscular complications either related to their underlying disease or as a result of an irAE. With regard to histological features, myopathy due to ICPI is characterised by necrosis with a large component of macrophage cells in clusters mimicking a pseudo-granulomatous pattern [89]. Myopathy due to ICPI appears in the muscle biopsy as a unique and characteristic pattern of inflammatory changes, far from any known inflammatory myopathy. When suspected, ICPI treatment discontinuation is recommended. High dose corticosteroid treatment is the first line treatment. The addition of IVIG or plasma exchange might be required in severe forms [63,96,97].

### 2.11. Kidney

ICPI can produce acute renal dysfunction (ARD) due to both its negative effect on the mechanisms of immunological tolerance, as well as the release of various cytokines (interleukin 6, TNF alpha, interferon gamma, CXCL10) and antibodies with direct toxic effect on renal tubular cells [98].

Initially, renal function deterioration was described in less than 1% of patients treated with ipilimumab, nivolumab and pembrolizumab. However, subsequent studies have confirmed a progressive increase in the frequency of grades 3–4 ARD [99,100]. In a study of 3695 patients under treatment with ICPI, Cortazar et al. [101] reported that the frequency of ARD was 2.2%, and 0.6% required dialysis. Subsequently, single centre studies [98] reported that the presence of ARD varied between 10–29% if all cases with significant elevation in creatinine levels were included. In addition to the alteration in renal function, the presence of haematuria (16%), eosinophilia (21%), hypertension (11%), pyuria (68%) and in a few cases, nephrotic syndrome, were also recorded. In 12 out of 13 patients in whom kidney biopsy was performed, the lesion pattern was acute tubulointerstitial nephritis (ATIN), the time of appearance of the lesion was 24–245 days after initiating treatment, 7 of 12 patients had extrarenal manifestations before the diagnosis of ARD (1 concomitant) and 9 of 10 cases treated with steroids presented complete or partial remission. These studies have also generated the hypothesis about the possible role of ICPI as agents that increase the risk of the appearance of immunoallergic ATIN associated with drugs widely used in clinical practice (non-steroidal anti-inflammatory drugs, antibiotics or proton-pump inhibitors, among others).

In the context of renal transplantation, the use of ICPI may increase the risk of developing acute rejection in up to 40–50% of patients [102].

### 2.12. Haematological

Haematological complications associated with ICPI treatment are rare. Autoimmune haemolytic anaemia (AIHA) has been associated with the use of ICPI including PD-1, PD-L1 and CTLA-4 directed antibodies. Although AIHA is an infrequent complication (<1%), it can be serious and immediate treatment with steroids is required together with ICPI discontinuation. In the case of insufficient response, the use of rituximab can be considered [103].

Other immune cytopenias, neutropenia, thrombocytopenia and even aplastic anaemia have also been described with the use of ICPI. Careful multidisciplinary evaluation of the risk-benefits of discontinuation of ICPI must be made according to the severity of the immune complication.

Hodgkin lymphoma patients undergoing allogeneic stem cell transplantation after salvage ICPI therapy have been reported to present a higher number of severe complications, particularly early acute graft-versus host disease [104]. Individualised risk evaluation of each patient is required until more data help to clarify such observations.

## 3. Critical Care of Severe irAEs

Although most irAEs related to ICPI are mild to moderate and are well managed in an outpatient setting, up to 20% of cases can be severe and even life-threatening (CTCAE grades III and IV) and lead to intensive care unit (ICU) admission [105].

The frequency of grades 3–4 irAE with anti-PD-1 and anti-PDL-1 seems to similar, being more common with CTLA-4 inhibitors (30%) and with combinations of these inhibitors (up to 55%). Moreover, the safety profile of ICPI varies among the different indications: melanoma has a higher risk of GI and skin irAE and lower frequencies of pneumonitis [106]. A recent systematic review of ICPI irAE that required ICU admission outside clinical trials showed that the most common events were perforated colitis or enterocolitis (17.6%), fulminant myocarditis (15.3%), polyradiculoneuritis (12.9%), pericarditis (10.6%) and MG (10.6%). Most of the reported cases concerned the anti-CTLA4 antibody ipilimumab in monotherapy (40.0%). The median time from ICPI initiation to ICU admission was 1.4 (0.2–16) months with a median number of two courses having been received [107].

The management of patients with severe irAE requires close collaboration among ICU specialists, organ specialists and cancer specialists as well as the adoption of standardised treatment protocols to ensure the best possible patient outcomes [105,106,108].

Discussions about the prognosis of the cancer patient and goals of care are mandatory. However, in cases in which ICPI induces organ failure, ICU admission of these patients should be considered due to their potential reversibility with treatment, at least for a time-limited trial [106], and this is even more important taking into account that high-grade toxicity seems to be associated with high tumoural response rates [109].

Treatment consists of supportive care and aetiological treatment. Regarding the first, mechanical ventilation and vasopressors are most often used, although renal replacement therapies or even extracorporeal life support may be needed [106,107]. Concerning aetiological therapy, for severe grades 3–4 irAE ICPI should be immediately discontinued and steroid treatment should be initiated as soon as possible. Whenever possible, it is of utmost importance to rule out alternative diagnoses before treatment, mainly infectious complications and cancer progression, although this may sometimes be challenging, and combined treatment must, in the meantime, be started.

## 4. Nursing and Immunotherapy

The role of nurses in the management of patients with indication of immunotherapy is key to patient education for the different treatments and for detecting AEs. The information transmitted to patients should be structured through therapeutic education programmes. These programmes facilitate knowledge in an organised manner, respecting individual needs and promoting the sensation of order and safety [110,111]. The aim of patient education is: (1) to provide the necessary knowledge to follow the treatments and maintain adherence, (2) to decrease anxiety and facilitate acceptance, (3) to include patients as active members of the team [112] and (4) to anticipate most common side effects and inform the patients of strategies to relieve symptoms.

It is necessary to have an easy access infrastructure that welcomes and provides confidence to patients. It should be fast and dynamic to respond in case of AEs. Nursing consultation, complemented with telephone contact, should be friendly and respond to clinical needs in order to provide agile response.

Nurses’ training is essential in order to detect possible ICPI complications. Nurses should know what to ask and how to respond, and should have experience and knowledge in the treatment as well as the underlying disease to thereby be able to differentiate treatment complications.

## 5. Conclusions

irAEs related to ICPI are relatively frequent and affect multiple organs and systems complicating patient management. The involvement of different medical specialties and nurses (working jointly with the oncologists), with in depth knowledge of the specific organ affected, increases our understanding of the pathogenesis of irAEs and, more importantly, assures the best personalised care and treatment.

## Figures and Tables

**Table 1:** Multidisciplinary approach to monitoring and treatment of immune-related adverse events.

Organ	Grade 1	Grade 2	Grade 3	Grade 4
Skin rash	-Physical examination and exclude other causes (viral rash, infection, other drug-induced rash); -Avoid skin irritants and sunlight; -Topical emollients and corticosteroids (mild strength) ± oral antihistamines for itching; -Continue ICPI treatment	-Consider dermatology consultation and skin biopsy; -Supportive management, as grade 1 + topical corticosteroides (moderate strength);	-Withhold ICPI treatment; -Management as grade 2 + oral prednisone (0.5–1 mg/kg); -For severe rash IV methylprednisolone (0.5–1 mg/kg) and convert to oral prednisone depending on response, wean over 2–4 weeks; -Resume ICPI at grade1/mild grade 2 after discussion with dermatologist;	-IV methylprednisolone (1–2 mg/kg); -Urgent dermatology consultation; -Discontinue ICPI treatment;
Colitis	-Rule out infections (see Section 2.2); -Symptomatic treatment with low-fibre diet, anti-diarrhoeals (loperamide), oral fluids; -Continue ICPI treatment;	-Rule out infections; -Consider colonoscopy (with biopsies); -Oral prednisone (1 mg/kg), tapered in 8–12 weeks; -If steroid-dependency consider IFX or VDZ; -GI consultation;-Withhold ICPI treatment;	-Rule out infections; -GI consultation; -Colonoscopy (with biopsies); -CT scan; -Hospital admission; -IV methylprednisolone (1 mg/kg); -Supportive measures; -If steroid-refractoriness (within 3–5 days) treat with IFX (vs. VDZ); -Discontinue ICPI treatment;	
Pneumonitis	-Symptomatic treatment; -Delay immunotherapy; - Monitoring;	-Withhold ICPI treatment; -Oral prednisone (1 mg/kg);	-Discontinue ICPI treatment; -IV methylprednisolone (2–4 mg/kg);	
Arthritis	-NSAID and acetaminophen; -Continue ICPI treatment;	-NSAID plus low doses of oral prednisone (< than 10 mg/day in tapering doses); -Continue ICPI treatment;	-Oral prednisone (0.5 mg/kg) in tapering doses; -Withhold ICPI treatment and resume upon symptom control; -If arthritis persists, consider MTX or LEF as maintenance treatment and steroid sparing agent;	-Consider IV methylprednisolone pulses (125 mg) followed by oral prednisone (0.5–1 mg/kg) in tapering doses; -Add MTX or LEF for persistent arthritis; -Discontinue ICPI treatment;
Renal	-Monitor renal function; -Check urine: sterile pyuria, WBC cast, proteinuria;	-Nephrology consultation; -Discontinue ICPI treatment; -Rule out other causes; -Monitoring;	-Nephrology consultation; -Renal biopsy; -IV methylprednisolone (1 mg/kg) if ATIN in the biopsy; -MMF if no response;	
Hepatitis	Monitoring	-Hepatology consultation; -Withhold ICPI; -Rule out other causes; -Monitoring;	-Hepatology consultation; -Check INR/albumin; -Liver biopsy; -Oral prednisone or IV methylprednisolone (0.5–1 mg/kg) if liver dysfunction *, severe inflammation in the liver biopsy or worsening (72 h) ;-AZA, MMF, or Tac if no response;	-Hepatology consultation; -Check INR/albumin; -Liver biopsy (should not delay treatment in hepatic encephalopathy or liver dysfunction *); -IV methylprednisolone (1 mg/kg/d); -AZA, MMF, or Tac if no response;
Neurological	-Delay immunotherapy; -Monitor symptoms;	-Central involvement: brain/spine MRI, CSF and antibody studies. Empiric antibacterial and antivirals; -Peripheral involvement: EMG, CK and antibody study; -Withhold ICPI treatment; -Neurology consultation; -Symptomatic treatment; -Oral prednisone (0.5–1 mg/kg);	-Central involvement: EEG, brain/ spine MRI, CSF and antibody studies. Empiric antibacterial and antivirals; -Peripheral involvement: EMG, CK and antibody study, vital capacity, cardiac monitoring to detect overlap syndromes; -Hospital admission, ICU monitoring or support; -Discontinue ICPI treatment; -Avoid medications that may worsen myasthenia; -IV methylprednisolone (1 gr × 3–5 days); -If partial or no improvement within one week IVIG (2 g/kg over 5 days) or plasma exchange; -Consider rituximab/ cyclophosphamide in refractory cases, paraneoplastic syndromes or proven antibody-mediated disease;	
Myopathy	-Monitor symptoms and muscle enzymes (aldolase and CK);	-Delay ICPI treatment; -Aldolase and CK. EMG, antibody study (specific and associated for myositis); -Whole body MRI and muscle biopsy; -Oral prednisone (0.5 mg/kg);	-Withhold ICPI treatment; -Aldolase and CK. EMG, antibody study (specific and associated for myositis); -Whole body MRI and muscle biopsy; -IV methylprednisolone(250 mg/day for 3 days) and then oral prednisone (0.5 mg/kg);	-Withhold ICPI treatment; -Aldolase and CK, EMG, antibody study (specific and associated for myositis); -Whole body MRI and muscle biopsy; -IV methylprednisolone (250 mg/day for 3 days) and then oral prednisone (0.5 mg/kg) plus IVIG or plasma exchange;
Blood disorders (Cytopoenia)	-Laboratory monitoring; -Continue ICPI treatment;	-Haematology consultation -Extended laboratory study ± bone marrow study; -Continue ICPI treatment;	-Withhold ICPI treatment; -Consider oral prednisone (1 mg/kg), haematopoietic growth factors, IVIG, rituximab or immunosuppressive drugs according to disorder type and severity;	-Blood disorders (Cytopoenia)

BSA: body surface area, DAE: dermatologic adverse event, AGPP: acute generalised pustular psoriasis, TEN: toxic epidermal necrolysis, DRESS: drug rash with eosinophilia and systemic symptoms, IV: intravenous, ICPI: immune checkpoint inhibitors, IFX: infliximab, VDZ: vedolizumab, GI: gastrointestinal; CT: computed tomography, NSAID: non-steroidalanti-inflammatory drugs, EEG: electroencephalogram; MTX: methotrexate, LEF: leflunomide, INR: International normalised ration, AZA: azathioprine, MMF: mofetil mycophenolate, Tac: tacrolimus, MRI: magnetic resonance imaging, CSF: cerebrospinal fluid, EMG: electromyography, CK: creatine kinase, ICU: intensive care unit, IVIG: intravenous immunoglobulin, WBC: white blood cells, ATIN: acute tubolintersticial nephritis. * Liver dysfunction is defined as bilirubin >2.5 mg/dL, coagulopathy (INR >1.5) and/or hepatic encephalopathy.

## Supplementary Materials

The following are available online at https://www.mdpi.com/2072-6694/12/11/3446/s1. Text S1. Endocrine irAEs, Table S1: Indications of immune check-point inhibitors, Table S2: Management of ocular toxicity. Table S3: Clinical manifestations and treatment of cardiac toxicity, Figure S1: Criteria for referring patients undergoing ICPI treatment for dermatological evaluation, Figure S2: Analytical monitoring at the beginning and during ICPI treatment for the detection of endocrine adverse events, Figure S3: Management of hypophysitis.
