# Supplementary material for: Multidisciplinary Clinical Approach to Cancer Patients with Immune-Related Adverse Events Induced by Checkpoint Inhibitors
Source: Cancers (Basel). 2020 Nov 19;12(11):3446. doi: 10.3390/cancers12113446 (PMC7699587; doi:10.3390/cancers12113446)
Supplement: Supplementary file 1 [file cancers-12-03446-s001.pdf]

Supplementary Material: Multidisciplinary Clinical Approach to Cancer Patients with Immune-Related Adverse Events Induced by Checkpoint Inhibitors

Maria-Carlota Londoño and Maria Reig on behalf of the RETOINMUNO Multidisciplinary Group

Text S1. Endocrine irAEs

Insulitis is a rare irAE (0.4%) which has only been described with anti-PD-1/anti-PDL-1 or in combination. It generally develops after the fourth dose of treatment, although it can be diagnosed after the interruption of the ICPI. Diabetic ketoacidosis is the form of presentation in 57% of cases: rapid onset of hyperglycemia, low levels of C-peptide with symptoms associated with insulinopenia (polydipsia, polyuria and weight loss) and vomiting and nausea if ketosis. Glycated hemoglobin (HbA1c) is usually slightly raised with fasting glucose >400 mg/dL, suggesting the rapid development of diabetes. Autoimmune antibodies are positive in 50% of cases and HLA-DR4 has been reported in 76% of patients [1]. Fulminant diabetes may be associated with exocrine deficit; pancreatic lipase should be measured. No image study is required for diagnosis. In ketoacidosis, intravenous insulin and hydro electrolytic replacement should be started without delay. Multiple day subcutaneous insulin treatment and diabetes education are the pillar of chronic treatment, as diabetes is generally permanent and the use of high doses of corticosteroids does not modify the course of the disease[2-4].

Immune-related pancreatitis is less seen irAE. The incidence is rare (CTLA4: 0.9–4%, PD-1: 0.5–1.6%; CTLA4 + PD-1: 1.2–13.3%) [5,6]. A metanalysis comparing ICPi with placebo or chemotherapy showed that both CTLA-4 inhibitors alone and combination treatment of nivolumab and ipilimumab could increase the risk of amylase or lipase elevation, but not significantly increased the risk of pancreatitis when compared with controls. Consistent with this data, some studies have proposed that routine monitoring of amylase/lipase in asymptomatic individuals is not recommended [5].

Adrenalitis is also infrequent (<1% in monotherapy, 4–8% in combination) and has been described in both anti-PD-1/anti-PDL-1 and anti-CTLA-4 from 2.5 to 5 months after the initiation of ICPI, although it can also appear after withdrawal [7,8]. The presentation may be a clinical picture of acute primary adrenal insufficiency (fatigue, weight loss, hypotension, nausea, vomiting and muscular pain) or only mild alterations including hyponatremia with/without hyperkalemia. In case of suspicion, cortisol assay and ACTH should be performed, and based on the severity of the clinical picture, parenteral or oral hydrocortisone treatment should be immediately started. Diagnosis can be ruled out if plasma cortisol is >500 nmol/L (18 mcg/dL) regardless of the time of day; levels <138 nmol/L (5 mcg/dL) with elevated ACTH confirm the diagnosis. For values between 128–500 nmol/L, a stimulation test with ACTH (adrenocorticotropic hormone)250 mcg test is recommended. Low aldosterone levels will help in the diagnosis of primary adrenal insufficiency and its distinction from ACTH secretion insufficiency if ACTH is not available. Anti-21 hydroxylase antibodies should be performed to examine an auto-immune cause and adrenal CT scan to rule out adrenal metastasis or hemorrhage. In case of acute adrenal insufficiency, stress doses of hydrocortisone and rehydration therapy should be begun as soon as possible. After improvement, the dose can be progressively reduced to replacement treatment with 15–30 mg/dL in 2-3 administrations. Fludrocortisone should be started at 50 mcg/d when the hydrocortisone dose is lower than 50 mg/24 h. Adrenalitis is generally permanent and will require chronic treatment and education of the patient to modify the dose in case of intercurrent disease and other physiological stress situations.

Table S1. Indications of immune check-point inhibitors.

Drug Name	Type of ICPI	Tumour Indication	Biomarker
Ipilimumab	CTLA-4 blocker	Melanoma<br>RCC	None<br>None
Pembrolizumab	PD-1 blocker	Melanoma<br>NSCLC<br>Hodgkin lymphoma<br>UC<br>ST MSI-High<br>GC cancer GEJ<br>HNSCC<br>Cervical cancer<br>Large B cell lymphoma<br>HCC<br>MCC<br>RCC<br>SCLC<br>ESCC<br>CRC<br>Endometrial carcinoma	Regardless of PD-L1<br>PD-L1 ≥ 50%; combined with CT if PD-L1 < 50% regardless of PD-L1 (1st line); regardless of PD-L1 (2nd line)<br>Regardless of PD-L1<br>PD-L1 CPS ≥ 10; regardless of PD-L1 if not eligible for platinum containing CT<br>MSI-H<br>PD-L1 CPS ≥ 1% (3rd line)<br>PD-L1 CPS ≥ 1% ± CT (1st line); PD-L1 TPS ≥ 50% (2nd line)<br>PD-L1 CPS ≥ 1%<br>Regardless of PD-L1<br>Regardless of PD-L1<br>Regardless of PD-L1<br>Combined with axitinib regardless of PD-L1<br>Regardless of PD-L1<br>PD-L1 CPS ≥ 10 % (2nd line)<br>MSI-H (1st line)<br>Combined with lenvatinib if no MSI-H/no dMMR
Nivolumab	PD-1 blocker	Melanoma<br>NSCLC<br>RCC<br>Hodgkin lymphoma<br>HNSCC<br>UC<br>ST MSI-High<br>CRC<br>HCC<br>SCLC<br>ESCC	Regardless of PD-L1<br>Regardless PD-L1<br>Regardless of PD-L1<br>Regardless of PD-L1<br>Regardless of PD-L1<br>Regardless of PD-L1<br>MSI-H<br>MSI-H/dMMR<br>Regardless of PD-L1<br>Regardless of PD-L1<br>Regardless of PD-L1
Cemiplimab	PD-1 blocker	CSCC	Regardless of PD-L1
Atezolizumab	PD-L1 blocker	UC<br>NSCLC<br>TNBC<br>SCLC<br>HCC	PD-L1 ≥ 5%<br>Combined with CT regardless of PD-L1 (1st line); Regardless of PD-L1 (2nd line)<br>Combined with CT if PD-L1 ≥ 1%<br>Combined with CT regardless of PD-L1<br>Combined with bevacizumab regardless of PD-L1
Avelumab	PD-L1 blocker	MCC<br>UC<br>RCC	Regardless of PD-L1<br>Regardless of PD-L1<br>Combined with axitinib regardless of PD-L1
Durvalumab	PD-L1 blocker	UC<br>NSCLC<br>SCLC	Regardless of PD-L1<br>PD-L1 ≥ 1%<br>Combined with CT regardless of PD-L1
Ipilimumab plus nivolumab	CTLA-4 blocker + PD-1 blocker	Melanoma<br>RCC<br>CRC<br>HCC<br>NSCLC	Regardless of PD-L1<br>Regardless of PD-L1<br>MSI-H/dMMR<br>Regardless of PD-L1<br>PD-L1 ≥1%; combined with CT regardless of PD-L1
T-VEC IT (talimogenelaherparepvec)	Oncolytic virus	Melanoma	Regardless of PD-L1 (recurrent disease)

Immune checkpoint inhibitors: ICPI; Renal cell carcinoma (RCC), Solid tumours (ST) with high microsatellite instability (MSI-H) or mismatch-repair deficiency (dMMR), Non-small cell lung

cancer (NSCLC), Urothelial carcinoma (UC), Gastric cancer (GC) and gastroesophageal junction adenocarcinoma (GEJ), Head and neck squamous cell carcinoma (HNSCC), Merkel cell carcinoma (MCC), Hepatocellular carcinoma (HCC), small cell lung cancer (SCLC), oesophageal cancer squamous cell (ESCC), endometrial cancer (EC), Colorectal cancer (CRC), chemotherapy (CT), triple negative breast cancer (TNBC), Cutaneous squamous cell cancer (CSCC), combined positive score (CPS), tumour proportion score (TPS), intratumoural (IT).

Table S2. Management of ocular toxicity.

Ocular Surface	Orbit and Ocular Adnexa	Optic Nerve	Anterior or Posterior Uveitis
Artificial tears<br>Topical cyclosporine<br>Topical corticosteroids	Systemic corticosteroids<br>Plasma exchange<br>IVIG	Systemic corticosteroids<br>Immunosuppressive therapy	Topical corticosteroids<br>Systemic Corticosteroids<br>Immunosuppressive therapy

IVIG: intravenous immunoglobulin.

Table S3. Clinical manifestations and treatment of cardiac toxicity.

irAE	Manifestation	ICPI Interruption	Cardiac Therapy	Immunosuppression
Myocarditis	-Asymptomatic or chest pain ± dyspnoea ± palpitations ± syncope<br>-Increased troponin levels<br>-Ventricular dysfunction (50%)<br>-Inflammation in CMR, PET-CT or biopsy	Usually recommended	-Intravenous diuretics + nitrates if pulmonary oedema<br>-Inotropes if cardiogenic shock.<br>-ACEI or A2R blockers + beta-blockers if LVEF<50%.<br>-Aldosterone antagonists, sacubitril-valsartan if LVEF<35%.<br>-Consider temporary use of LV assist devices if refractory cardiogenic shock	-IV methylprednisolone (500-1000 mg/d) until clinically stable followed by oral prednisone (1 mg/kg) and progressive weaning.<br>-Alternatives: MMF, IFX, anti-thymocyte globulin or IVIG
Advanced atrioventricular block.	-Asymptomatic or dyspnoea ± syncope<br>-ECG with second degree mobitz 2 or complete atrioventricular block	Yes	Pacing	Consider IV methylprednisolone if suspicion of associated myocarditis (increased troponin levels, oedema in CMR).
Acute pericarditis	Chest pain (it increases with inspiration and supine position)	Yes, consider reintroduction	NSAID and colchicine	IV methylprednisolone with progressive weaning
Cardiac tamponade	-Usually associated with chest pain and right heart failure<br>-Hypotension.<br>-Tachycardia<br>-May cause multi-organ failure.	Yes, consider reintroduction after pericardiocentesis	Pericardiocentesis	-Consider methylprednisolone with progressive weaning.
Myocardial infarction	- Chest pain<br>- Dyspnoea.	Yes, consider reintroduction after 30 days if clinically stable	Consider urgent coronary angiogram	Consider methylprednisolone if suspicion of vasculitis
Systolic dysfunction without inflammation	-Asymptomatic or dyspnoea, asthenia, congestive signs (crackles, oedema, jugular ingurgitation).	Yes, consider reintroduction if normal ventricular function recovered and myocarditis excluded	-Follow-up with imaging, particularly echocardiography and CMR<br>-Intravenous diuretics + nitrates if pulmonary edema<br>-Inotropes if cardiogenic shock<br>-ACEI or A2R blockers + beta-blockers if LVEF < 50%<br>-Aldosterone antagonists, sacubitril-valsartan if LVEF < 35%.	Not indicated

Takotsubo syndrome	-Chest pain<br>-May cause dyspnoea and even cardiogenic shock.	Yes, consider reintroduction if normal ventricular function recovered and myocarditis excluded	-Coronary angiogram<br>-Follow-up with imaging, particularly echocardiography and CMR<br>-If heart failure, follow same pharmacologic treatment as in systolic dysfunction without inflammation	No evidence recommending its use
Atrial fibrillation	-Palpitations<br>-May be asymptomatic	Yes, consider reintroduction if clinically stable and myocarditis excluded	-Strategy of rhythm vs. frequency control (according to clinical guidelines).<br>-Anticoagulation based on CHA_2DS_2VASc	No evidence supporting its use
Ventricular arrhythmias	-Palpitations<br>-Chest pain<br>-Sudden death	Yes	Electrical/ pharmacological cardioversion or defibrillation	Consider methylprednisolone if suspicion of associated myocarditis (increased troponin levels, edema in CMR)
Elevated natriuretic peptides in asymptomatic patient	-Asymptomatic.<br>-Can be associated with congestive signs (oedema, jugular ingurgitation)	Continue if no evidence of ventricular systolic dysfunction or myocarditis	Complete anamnesis, physical exam, troponin, ECG, echocardiography and consider CMR	Not indicated
Elevated troponin in asymptomatic patient.	Asymptomatic	Yes, until exclusion of coronary disease or myocarditis	ECG, serial troponin, echocardiography, CMR. Consider coronary angiogram	Not indicated

irAE: immune-related adverse event; CMR: cardiac magnetic resonance, PET-CT: Positron emission tomography–computed tomography, ACEI: angiotensin converting enzyme inhibitors, A2R: angiotensin II receptor, LVEF: left ventricular ejection fraction, LV: left ventricle;IV intravenous; MMF: mofetil mycophenolate, IVIG: intravenous immunoglobulin, NSAID: non-steroidal antiinflammatory drug; IFX: infliximab, ECG: electrocardiogram, CHA_2DS_2VASc: score to predict thromboembolic risk in patients with atrial fibrillation.

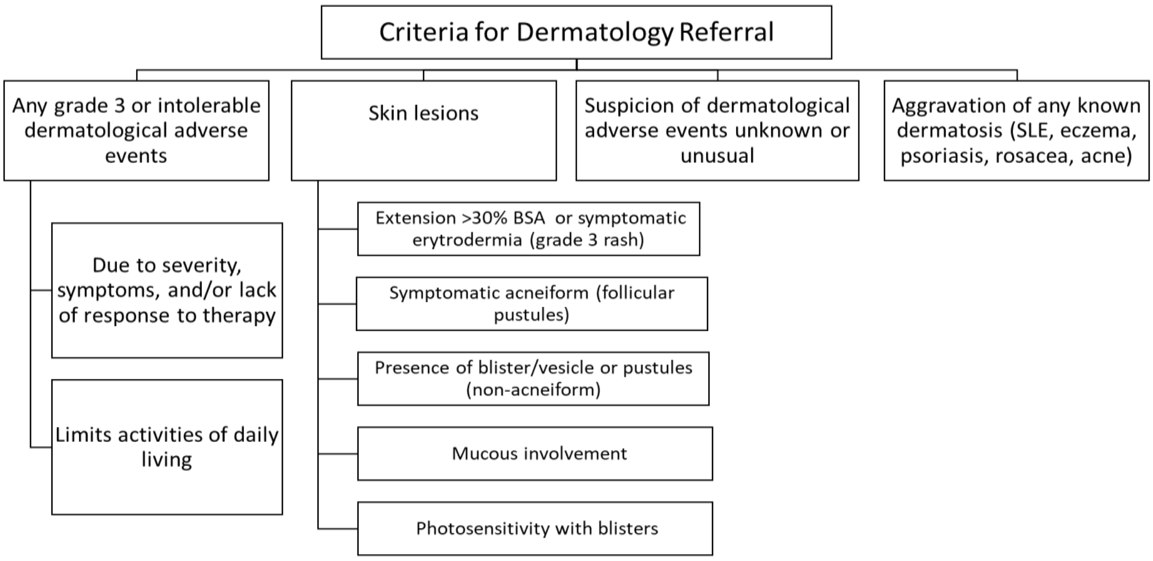

Figure S1. Criteria for referring patients undergoing ICPI treatment for dermatological evaluation.

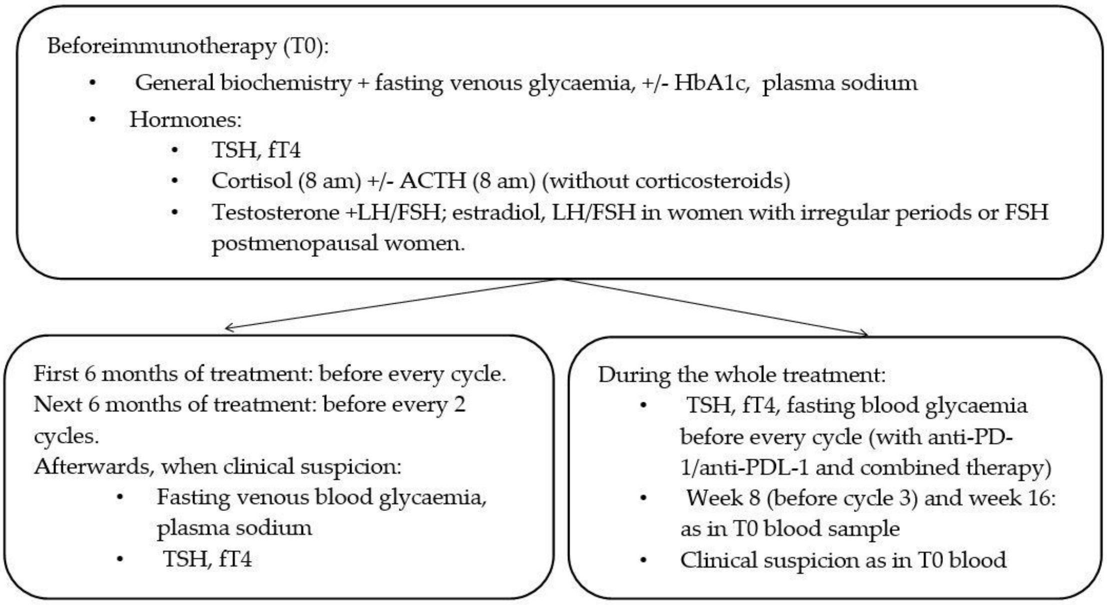

Figure S2. Analytical monitoring at the beginning and during ICPI treatment for the detection of endocrine adverse events. T0: before immunotherapy; HbA1c: glycated haemoglobin; TSH: thyroid stimulating hormone; fT4: free tyroxine; LH: luteinizing hormone; FSH: follicle stimulating hormone.

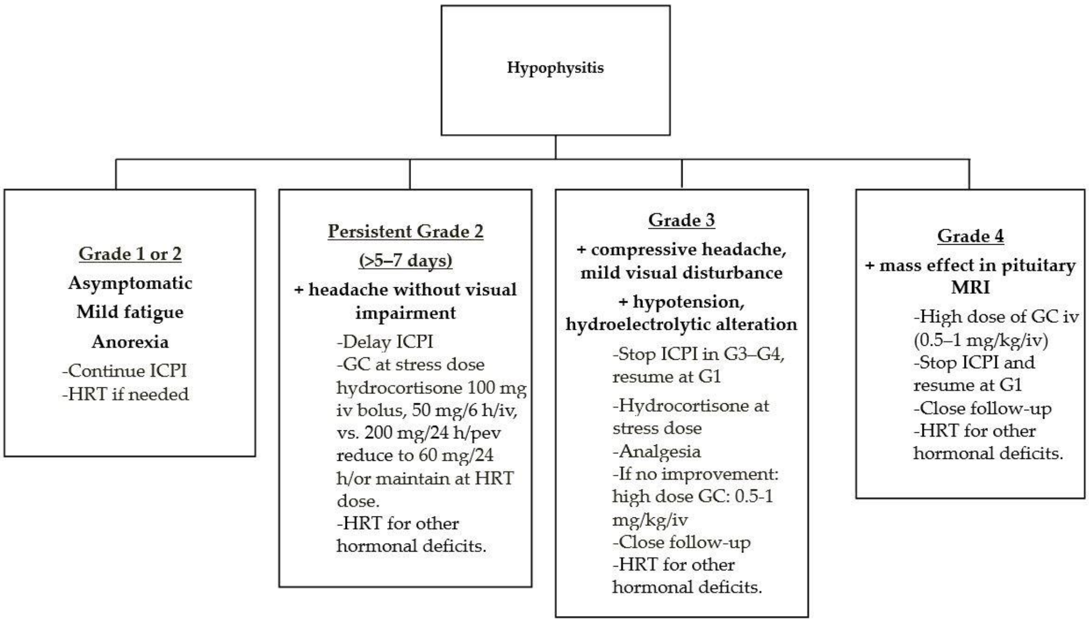

Figure S3. Management of hypophysitis.

References

1. Stamatouli, A.K.; Quandt, Z.; Perdigoto, A.L.; Clark, P.L.; Kluger, H.; Weiss, S.A.; et al. Collateral Damage: Insulin-Dependent Diabetes Induced With Checkpoint Inhibitors. *Diabetes* **2018**, *67*, 1471-1480. doi: 10.2337/dbi18-0002
2. Castinetti, F.; Albarel, F.; Archambeaud, F.; Bertherat, J.; Bouillet, B.; Buffier, P.; Briet, C.; Cariou, B.; Caron, P.; Chabre, O.; et al. French Endocrine Society Guidance on endocrine side effects of immunotherapy. *Endocr.-Related Cancer* **2018**, *26*, G1–G18, doi:10.1530/ERC-18-0320.

3. Chang, L.-S.; Barroso-Sousa, R.; Tolaney, S.M.; Hodi, F.S.; Kaiser, U.B.; Min, L. Endocrine Toxicity of Cancer Immunotherapy Targeting Immune Checkpoints. *Endocr. Rev.* **2019**, *40*, 17–65, doi:10.1210/er.2018-00006.
4. Tan, M.H.; Iyengar, R.; Mizokami-Stout, K.; Yentz, S.; MacEachern, M.P.; Shen, L.; Redman, B.; Gianchandani, R. Spectrum of immune checkpoint inhibitors-induced endocrinopathies in cancer patients: a scoping review of case reports. *Clin. Diabetes Endocrinol.* **2019**, *5*, 1–21, doi:10.1186/s40842-018-0073-4.
5. Su, Q.; Zhang, X.-C.; Zhang, C.-G.; Hou, Y.-L.; Yao, Y.-X.; Cao, B. Risk of Immune-Related Pancreatitis in Patients with Solid Tumors Treated with Immune Checkpoint Inhibitors: Systematic Assessment with Meta-Analysis. *J. Immunol. Res.* **2018**, *2018*, 1–9, doi:10.1155/2018/1027323.
6. George, J.; Bajaj, D.; Sankaramangalam, K.; Yoo, J.W.; Joshi, N.S.; Gettinger, S.; Price, C.; Farrell, J.J. Incidence of pancreatitis with the use of immune checkpoint inhibitors (ICI) in advanced cancers: A systematic review and meta-analysis. *Pancreatol.* **2019**, *19*, 587–594, doi:10.1016/j.pan.2019.04.015.
7. Cukier, P.; Santini, F.C.; Scaranti, M.; O Hoff, A. Endocrine side effects of cancer immunotherapy. *Endocrine-Related Cancer* **2017**, *24*, T331–T347, doi:10.1530/erc-17-0358.
8. Paepegaey, A.-C.; Lheure, C.; Ratour, C.; Lethielleux, G.; Clerc, J.; Bertherat, J.; Kramkimel, N.; Groussin, L. Polyendocrinopathy Resulting From Pembrolizumab in a Patient With a Malignant Melanoma. *J. Endocr. Soc.* **2017**, *1*, 646–649, doi:10.1210/js.2017-00170.

Publisher's Note: MDPI stays neutral with regard to jurisdictional claims in published maps and institutional affiliations.

© 2020 by the authors. Licensee MDPI, Basel, Switzerland. This article is an open access article distributed under the terms and conditions of the Creative Commons Attribution (CC BY) license (http://creativecommons.org/licenses/by/4.0/).
